# Dynamics of Bioactive Compounds during Spontaneous Fermentation of Paste Obtained from *Capsicum* ssp.—Stage towards a Product with Technological Application

**DOI:** 10.3390/plants11081080

**Published:** 2022-04-15

**Authors:** Csaba Balázs Kádár, Adriana Păucean, Elemér Simon, Dan Cristian Vodnar, Floricuța Ranga, Iulian Eugen Rusu, Vasile-Gheorghe Vișan, Simona Man, Maria Simona Chiș, Georgiana Drețcanu

**Affiliations:** 1 Department of Food Engineering, Faculty of Food Science and Technology, University of Agricultural Sciences and Veterinary Medicine of Cluj-Napoca, 3–5 Mănăștur Street, 400372 Cluj-Napoca, Romania; balazs-csaba.kadar@usamvcluj.ro (C.B.K.); iulian.rusu@usamvcluj.ro (I.E.R.); simona.man@usamvcluj.ro (S.M.); simona.chis@usamvcluj.ro (M.S.C.); 2 Department of Food Science, Faculty of Food Science and Technology, University of Agricultural Science and Veterinary Medicine of Cluj-Napoca, 3–5 Calea Mănăștur, 400372 Cluj-Napoca, Romania; simon.elemer@usamvcluj.ro (E.S.); dan.vodnar@usamvcluj.ro (D.C.V.); floricutza_ro@yahoo.com (F.R.); georgiana.dretcanu@stud.ubbcluj.ro (G.D.); 3 Faculty of Food Science and Technology, Institute of Life Sciences, University of Agricultural Sciences and Veterinary Medicine of Cluj-Napoca, 3–5 Mănăștur Street, 400372 Cluj-Napoca, Romania; 4 Department of Fundamental Sciences, Faculty of Animal Science and Biotechnologies, University of Agricultural Sciences and Veterinary Medicine of Cluj-Napoca, 3–5 Mănăștur Street, 400372 Cluj-Napoca, Romania; gelu.visan@yahoo.com

**Keywords:** *Capsicum* spp., spontaneous fermentation, phenolic compounds, capsaicinoids, enriched products

## Abstract

Six cultivars of chili (Cherry, Bulgarian Chilli, Cayenne, Fatalii, Habanero, and Carolina Reaper) from two species (*Capsicum annuum* and *Capsicum chinense*) have been studied. Anaerobic, spontaneous fermentation of pure chili paste was conducted for 21 days at 20 °C. The unfermented (UCP) and fermented chili pastes (FCP) were both subjected to physicochemical and microbiological characterization consisting of capsaicinoid, ascorbic acid, short-chain organic acids, phenolic compounds, and simple sugars analysis. Cell viability for Lactic Acid Bacteria (LAB) and *Leuconostoc* was determined before and after fermentation. Results indicate that capsaicinoids are very stable compounds, as notable differences between unfermented and fermented samples could not be seen. Carolina Reaper and Fatalii cultivars were amongst the most pungent, whereas Cherry, Cayenne, and Bulgarian types were low to moderate in pungency. Average loss of total ascorbic acid was 19.01%. Total phenolic compounds ranged between 36.89–195.43 mg/100 g for the fresh fruits and 35.60–180.40 mg/100 g for the fermented product. Losses through fermentation were not significant (*p* < 0.05). Plate counts indicated low initial numbers for LAB in the fresh samples, values ranging between 50–3700 CFU/g (colony-forming units). After fermentation, day 21, concentration of LAB (3.8 × 10^6^–6.2 × 10^8^ CFU/g) was high in all samples. Fermented chilies paste with enhanced biochemical and bacterial properties might further be used in the technology of vegetable (brining) or meat (curing) products, processes that generally involve the fermenting activity of different microorganisms, especially (LAB). Thus, the purpose of this research was the investigation of biochemical and microbial transformations that naturally occur in fermented chilies with a future perspective towards technological applications in cured meat products.

## 1. Introduction

Regardless of ethnicity, tradition, geography, or economic well-being, chilies are to be found all across the globe in almost every single household. That might be a reason for their intense scientific observations through many domains: agriculture, food science, biotechnology, medical sciences, and many more [1,2,3,4,5].

There is no net differentiation between chilies regarded as common food or spice, each culture uses them according to their will and tradition.

The *Capsicum* genus, within the *Solanaceae* family, includes many wild species (approx. 37), but only 5 domesticated that are more generally cultivated in different regions of the globe: *C. annuum*; *C. chinense*; *C. frutescens*; *C. baccatum*, and *C. pubescens* [6,7]. These five species include more than 200 cultivars, distributed unevenly across many countries. *C. annuum* is generally more common and includes fruits of multiple sizes and shapes, whereas *C. chinense* and *C. frutescens* are considered to be phylogenetically related and contain more exotic cultivars and types. *C. chinense* is also regarded as the species with some of the most pungent varieties to be known: Habanero, Carolina Reaper, Scotch Bonnet, Trinidad Scorpion, Ghost pepper, etc. [8]. In contrast, *C. baccatum* and *C. pubescens* are more specific to the continent of South America, mainly to the Andean region (Peru, Bolivia, etc.) [8,9].

Amongst many particular characteristics, chilies present a few common properties. There is a group of chemical substances (alkaloids) found unevenly distributed amongst different cultivars, causing moderate or highly burning sensations [10], known generically as capsaicinoids [9,11]. These molecules, amongst others, are primary biomolecules characteristic to chilies. They are synthesized exclusively in the placenta of the chili fruit, between 20–30 days after flowering, the process being carried out through the remaining period until full development of the fruits [12].

Biosynthesis of capsaicin includes two major pathways: the first one involves the synthesis of vanillylamine from phenylalanine, whereas the second one involves valine as the substrate, leading to the formation of 8-methyl-6-noneonil-CoA [13,14,15]. Condensation between the two molecules is catalyzed by the acyltransferase, an enzyme encoded by the gene *AT3*, also known as *Pun1* gene [15,16]. The absence of the enzyme in sweet variety pepper fruits interrupts the reaction chain, leading to the accumulation of vanillylamine in the fruit tissue [17].

Capsaicin and its derivatives can present strong fluctuations between species and cultivars, their concentration being strongly correlated with genetics and growing conditions [8].

Capsaicin and chili consumption is highly debated amongst scientists regarding their effects on health. The burning sensation, induced by capsaicinoids, starts by the cleavage of the molecule to the TRPV1 (vanilloid) receptors on the surface of nerve cells, and works in the same way as in the case of mechanical and thermal stimulation of nociceptors, found mainly in the peripheral nervous system [18].

Ascorbic acid (vitamin C) is another bio-compound, specific to peppers in general, that plays a key role in the neutralization of free radicals, thereby working as a protective agent against cellular damage [19,20]. Besides these, ascorbic acid participates in important physiological processes, including collagen synthesis, iron absorption, epigenetic regulations, immune system stimulation, with daily requirements being between 75–90 mg [21]. Chili peppers represent a good source of vitamin C, having a content between 150–180 mg/100 g fresh weight (FW) [22]. Ascorbic acid usually is found in two forms: the reduced form (ascorbic acid) and an oxidized form (dehydroascorbic acid). The second one, although still reducible to its original form by enzymatic catalyzers (glutathione, NADH, or NADPH), is quite unstable under physiological conditions [23,24,25]. This means that the dehydroascorbic acid can also be oxidized to 2,3-diketogulonic acid, the first compound in a series of reactions that leads to the irreversible oxidation of the compound [24]. Dehydroascorbic acid, however, can be stabilized in aqueous medium at low pH levels (pH = 2–4) [26]. Amongst the reducing agents for dehydroascorbic acid, there are cysteine, H_2_S, dymercapto-1-propanol, or different thiols [27].

Some studies indicate that field crops contain higher doses of vitamin C than those grown under protective conditions (greenhouse, foils, etc.). Summer crops are also richer in vitamin C (129–132 mg/100 g) than early crops or protected crops, indicating the importance of natural and organic farming in plant growing [28,29]. Green chilies tend to contain higher concentrations of ascorbic acid, a tendency that is kept just until full development, moderate decrease being observed at the final stage [30]. Intensely colored varieties (green, red, orange, and brown) also contain more ascorbic acid than white, purple, or black colored varieties [31].

Phenolic compounds (including flavonoids) are also representative biomolecules for chili peppers [32]. These aromatic compounds usually contain -OH (hydroxyl) groups attached to the phenol rings. There are more than 8000 different compounds, with molecular masses ranging from a few Da to >30 kDa. Most of them present antioxidant activity, whereas others can also present antimicrobial activity [33]. The antioxidant effect in phenols is dependent on the presence of the hydroxyl group, solubility of the compound and steric effect [34]. Studies indicate that quercetin and luteolin are the main flavonoids associated with peppers, representing 41% of total flavonoids identified [6,35]. Some theories suggest that phenolic compounds play a major role in the antioxidative protection of the fruits in the maturation process. This can explain the decrease of the compound in the late stages of ripening [6]. Amongst other phenolic compounds, peppers include vanillic acid, caffeic acid, *p*-coumaric acid, *p*-hydroxybenzoic acid, and ferulic acid. Most of the flavonoids found in peppers are glycosides or glycans of quercetin, myricetin, luteolin, apigenin, or kaempferol [35].

Spontaneous fermentation is a very primitive thus simple way, used for vegetable preservation throughout the world, involving generally minimal costs. Processes initiated by strong enzymatic action of bacteria over complex nutrients (proteins, starches, lipids, cellulose, etc.) lead to hydrolytic decomposition of the major molecules to smaller, biologically assimilable substances, most of them presenting beneficial effects over health (vitamins, anti-bacterial substances, organic acids, aromatic compounds, etc.) [36]. These processes are carried out besides strong CO_2_ generation and the formation of organic acids, mainly lactic acid. Acid formation leads to the drop of pH (<4), creating optimum conditions for bacterial growth inhibition and molecular stability for specific molecules (e.g., vitamin C) [37].

The process is usually carried out by LAB species and yeasts, identifiable on raw material (indigenous microflora), that present good adaptation and proliferation capabilities on the substrate [38]. Bacterial metabolism creates a series of biochemical transformations in the brine or the product, leading to quality improvement and shelf-life stabilization [39]. *Lactobacillus plantarum* is considered to be the predominant microorganism in vegetable fermentation, which is recently becoming a subject of more interest. Exopolysaccharides produced by these bacteria can present beneficial effects on rheological properties of fermented food [40]. Studies indicate beneficial, probiotic activity of different *L. plantarum* strains, as surface proteins detected on the exterior of cells can increase adhesion to epithelium [41]. Other findings underline the fact that *L. plantarum* metabolites (lactic acid, citric acid, etc.), show strong antifungal activity [42]. Cell free supernatants of LAB were shown to inhibit aflatoxin production by 91% [43]. *L. plantarum* strains are also highly adaptable to extreme conditions: high acidity, bile toxicity, etc. These are all important issues regarding food technology, food safety, and future techniques for product development.

From the technological point of view, some LAB species, found in fermented vegetables, might serve as mixed starter cultures for other products. However, in many cases these starters need to fulfill specific conditions: proteolytic activity, good survival in high saline concentration, high amounts of acid production, etc. [38].

The aim of the present study was to investigate the potential effect of spontaneous fermentation on bioactive compounds contained by different pepper cultivars. For this reason, six cultivars of chili, related to two different species (*C. annuum* and *C. chinense*) were spontaneously fermented for 21 days at 20 ± 1 °C. Bio-compounds and LAB cell viability were investigated in both fresh and fermented forms. Fermented chili mash might serve as a carrier for natural LAB starter cultures in vegetable fermentation or cured meat products. It may also represent a new approach in food technology, which might result in cost reduction and better sensory properties of end products that resemble characteristics of traditional foods. This possibility is currently investigated in a parallel research, that characterizes the adaptability and dynamics of microbial populations (LAB) in dry aged meat products (salami), with different sources of fermenting bacteria: spontaneous, starter culture, fermented chili powder, and fermented chili paste. The current article, however, discusses the transformations that can occur during fermentation of chilies, with strong technological implications in meat curing, e.g., effect of fermented chilies on pH reduction in minced meat, initiation and rapid growth of LAB, antioxidant activity of different pepper compounds, inhibition of pathogens by capsaicinoids and polyphenols, and overall stability of end products.

## 2. Results and Discussion

### 2.1. Moisture and Fruit Components Content

In order to evaluate relationship between different pepper types, moisture content and proportion of different structural parts were evaluated. This is important due to the fact that the quality of the paste increases with the increase of placenta fraction and reduced moisture content.

A significant interaction is observed in the proportion of the partitions (*p* < 0.05). These results are logical, since variation amongst pepper fruits related to the same cultivar is high. In these conditions, a significant increase in seeds, leads to the decrease of pericarp tissue: for example, average seed content for Cherry peppers is 12.67 ± 1.28%, average placenta content is 11.91 ± 0.25%, whereas pericarp content is 75.42 ± 1.47% as shown in Table 1.

In contrast, Bulgarian type peppers show very low seed content (2.82 ± 1.04%), contrasted with high pericarp content (90.68 ± 3.70%). These correlations can be identified throughout all pepper samples, and might give an insight on the partition of different biomolecules in pepper fruits. It is important to highlight that pericarp is the most abundant part in glycosidic compounds and terpenoids, placental tissue is the richest part in capsaicinoid-related compounds, alkaloids, and tocopherols, while seeds contain fatty acids and saponins [44].

Regarding moisture content, it seems that a universal increase is evident through fermentation for all samples. Differences are statistically significant (*p* < 0.05), both between cultivars, and fresh and fermented samples within the same cultivar. Average moisture content for Cherry-type pepper increases from 85.48 ± 2.06% to 86.96 ± 0.59% during a 21-day fermentation. For Cayenne pepper, there is an increase from 88.14 ± 1.02% to 90.34 ± 1.25%. A general increase in humidity can be observed among all samples within the range of 0.76–3.66%, as it is shown in Table 2.

### 2.2. Capsaicinoids

Four capsaicinoids were determined: capsaicin, dihydrocapsaicin (DHC), nordihydrocapsaicin (n-DHC), and homodihydrocapsaicin (h-DHC). Capsaicin and DHC were the two major compounds. As expected, *C. chinense* cultivars were the most pungent [45], whereas *C. annuum* includes mostly low or moderately pungent varieties. As it is shown in Table 3, total capsaicinoid content ranged between 0.02% (Bulgarian peppers and Cayenne peppers) and 1.80% (Carolina Reaper) FW. Differences between cultivars are statistically significant (*p* < 0.001). In addition, differences are evident amongst the same cultivars in the fresh and fermented forms (*p* < 0.05). Stability of capsaicinoids is observed through fermentation, total content of capsaicinoids being easily increased by the process, probably due to better extraction and maceration of the product. This trend can be observed in all samples. Carolina Reaper shows an increase of total capsaicinoids from 16.56 ± 0.31 mg/g (fresh) to 18.00 ± 0.08 mg/g (fermented). From the mild varieties, Cherry peppers show an increase from 0.48 mg/g (fresh) up to 0.63 mg/g (fermented).

Do, 2017 [46], also identified Carolina Reaper as a very pungent pepper type, underlining higher capsaicinoid concentrations in whole peppers (seeds included). Whole Carolina Reaper peppers contained 13% more total capsaicinoids than those without seeds and partitions. His results on capsaicin content indicate values of 100.00 ± 5.00 mg/g dry weight (DW), quite close to our results 9.57 ± 0.27 mg/g (FW).

Distribution of major and minor capsaicinoids, however, presents a variable trend. Capsaicin represents the primary major capsaicinoid, counting values between 47.48–59.74% for the fresh samples. These results are similar to the results of Dueland et al. [47], who determined capsaicin in the range of 31% to 71% of total capsaicinoids, and also contradicting other studies which indicate that capsaicin might count for more than 90% of total content [16].

**Figure 1:**
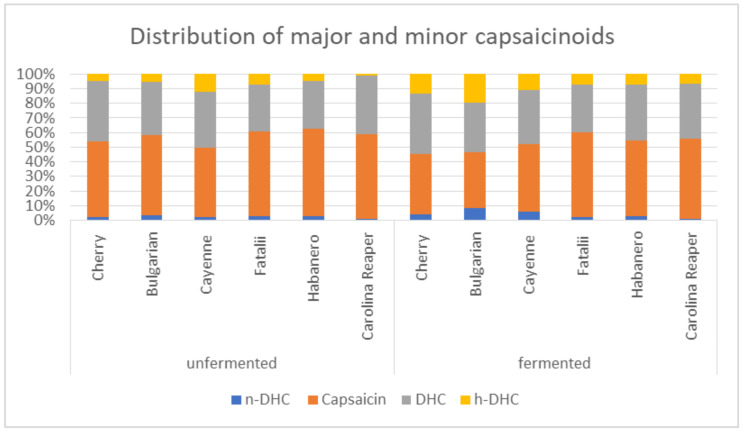
Mean distribution (%) of major and minor capsaicinoids in the fresh and fermented chili samples.

All capsaicinoids present an increasing trend through fermentation although the different ratios change during this time, as is evident in Figure 1. Capsaicin levels drop slightly in relation with the other compounds, reaching values between 38.05–57.73% in the FCP. DHC presents more constant values, results indicating an almost insignificant drop from values ranging between 32.24–41.47% (fresh peppers) to values in the range of 32.45–40.97% (fermented peppers). These slight changes are also confirmed in other studies [48]. Average distribution of capsaicin for the fresh samples is 54.93% with a decrease to 48.27% after 21 days. Dihydrocapsaicin stands for 36.77% of total capsaicinoids with an average of 36.68% after fermentation.

Minor capsaicinoids are represented by n-DHC and h-DHC. Average content of n-DHC was 2.38% in the fresh paste, with an average increase to 4.16% in the fermented samples. The same tendency was observed for h-DHC, which increased from 5.91% to 10.88%. Capsaicin tends to show higher values in the case of *C. chinense* cultivars, and slightly lower values for *C. annuum* [45].

In order to evaluate the pungency of the samples, expressed in Scoville Heat Units (SHU) based on the Scoville scale, results of capsaicinoids content (parts per million—ppm) were multiplied with standard coefficient values, specific for the pungency of the individual capsaicinoids, results being correlated to dry weight, as described by Dueland. Coefficients were used as follows: capsaicin (×16); DHC (×16); n-DHC (×9.1) and h-DHC (×8.6). Results shown in Table 4 indicate statistically significant differences amongst species, cultivars and different forms (fresh/fermented) within the same cultivars (*p* < 0.05).

Results show that the Bulgarian chilies are the mildest amongst the examined samples with scores of 18,844 ± 723 SHU for the fresh and 31,835 ± 724 SHU for the fermented paste. Carolina Reaper showed maximum values, ranging between 1.99 million ± 55,578 SHU (for the fresh sample) and 2.54 million ± 35,263 SHU for the fermented samples (DW). These values are comparable with the results of Dueland. In his studies, he obtained a pungency level of 1,046,000 ± 3400 SHU for the Carolina Reaper, 247,000 ± 25,000 SHU for Habanero and 17,000 ± 700 SHU for Fatalii.

During fermentation, capsaicinoids become more available through maceration and self-extraction. Stability of capsaicinoids during chili fermentation is also evidenced in a study regarding chili fermentation in wooden and plastic barrels [48]. As it can be observed fermentation leads to better maceration and extraction of capsaicinoids from the capsaicin glands, thereby SHU values increased by 28% in the case of Cherry, 41% for Bulgarian, 48% for Cayenne, 32% for Fatalii, 25% for Habanero, and 21% for Carolina Reaper. This may explain why pepper sauces tend to be more pungent than the peppers they are made of, excepting cases when they are diluted.

Capsaicin content of chilies is quite variable even throughout the same types or cultivars, as many endogenic or exogenic factors act selectively in capsaicinoids accumulation [16]. This might also be evidenced in the pungency of Fatalii-type peppers. In our study we obtained a pungency of 406,524 ± 17,511 SHU for the fresh sample, whereas Dueland (2016) obtained 17,000 ± 700 for the same type grown in Denmark [47]. Ana Carolina de Aguiar, (2016) [49] also obtained similar results for the Fatalii pepper (approx. 17,000 SHU), although in her case capsaicinoids were determined on peeled samples without seeds (results reported for fresh weight) whereas we used whole peppers. Dueland, also reported his results to the dry weight, therefor his results might be seen contradictory as Fatalii-type peppers are considered very hot-type peppers (400,000–500,000 SHU).

### 2.3. Ascorbic Acid

Statistical analysis shows significant differences regarding ascorbic acid content between cultivars and amongst fermented and unfermented samples within the same types (*p* < 0.01). This is also underlined by literature, as according to Hernández [9] ascorbic acid can present a wide range of expectancy (20–247 mg/100 g), induced by many factors, such as: genotype, maturity stage, harvesting period, climate factors, etc. [50].

In our case, Cayenne pepper showed the highest average amount for total ascorbic acid (134.58 ± 0.93 mg/100 g fresh and 107.81 ± 2.58 mg/100 g fermented). These values are slightly higher than those of Howards (63.24 mg/100 g), in case of fresh Cayenne-type fruits. Zamljen, (2021) [51], also obtained maximum levels for ascorbic acid in the pericarpal tissue of Cayenne peppers, amongst 21 cultivars investigated. Fluctuation of such values is totally acceptable, as results are highly influenced by many factors (growing conditions, sample preparation, etc.)

Values might also drop in case of peppers grown under protective conditions (green house) [22,28]. Also, in case of net shading, the color of the material might influence vitamin C content [28].

Average values are similar to those of Sidonia Martinez (2015) [32]. In her study regarding variation of ascorbic acid through maturation, she found values ranging between 106.05 ± 6.30 mg/100 g for green peppers and 148.94 ± 5.06 mg/100 g for red peppers. Slightly higher values in the case of this study can be explained by sample preparation. In this study tests were conducted over the pericarp (peduncles and seeds discarded) [32], whereas in our study whole pepper fruits were used (seeds included). This might confirm that vitamin C is more specific to the pericarpal tissue of the fruit, whereas other parts can lead to a decrease of ascorbic acid content if taken into account.

Studies show a relatively increasing trend in vitamin C accumulation towards the final stages of ripening [31], although this may not be regarded as a general trend. Some studies indicate that moderate water deficiency tend to increase vitamin C and capsaicinoid content [52].

In the case of Habanero chilies, we obtained mean values for total ascorbic acid content of 78.46 ± 2.84 mg/100 g. A study conducted by Ana Flávia Teodoro, (2013) [53] on Habanero pepper accessions showed quite similar results. Her findings show values in the interval of 51.1–129.8 mg/100 g [33]. These experiments were also conducted on whole fruits (seeds included). Values for the red Habanero (the same as we used) were very close to our results (76.7 mg/100 g and 85.0 mg/100 g).

Total average losses in vitamin C content after fermentation represent 19.89%. Average values of ascorbic acid and dehydroascorbic acid and total losses after fermentation are shown in the Table 5.

Lowest levels in vitamin C content were observed in Carolina Reaper-type peppers with an average amount of 56.66 ± 1.32 mg/100 g fresh and 43.16 ± 2.50 mg/100 g fermented with average losses of 23.82% over a period of 21 days fermentation. Average losses of total ascorbic acid are 19.01% with minimums occurring in the case of Bulgarian chili (12.43%) and maximum for Carolina Reaper (23.82%). Correlation between pungency and ascorbic acid content for fresh fruits is not evident.

Average losses of ascorbic acid (the reduced form) are 11.01% with minimum in the case of Habanero peppers (3.08%) and maximum in the case of Cayenne (21.65%). These average losses, however, are lower in comparison with dehydroascorbic acid (the oxidized form), which includes total average losses of 23.03% with minimum values for Cayenne (15.58%) and higher percentage for Carolina Reaper (29.31%).

Ascorbic acid is relatively more stable as a molecule than its primary oxidized form (dehydroascorbic acid). Although the two molecules are interconvertible, dehydroascorbic acid can more easily be further oxidized to its irreversible form (2,3-diketogulonic acid). Stabilization of dehydroascorbic acid occurs in aqueous medium at pH < 4 Deutsch [24]. Under present circumstances, these values can be achieved in 3–5 days. This may explain the relatively moderate amount of dehydroascorbic acid degradation in the first stage of the process. Rapid reduction of pH, however, contributes to the stabilization of the molecule and prevents further losses, contributing to the preservation of high amounts of the molecule. It is also not evident if dehydroascorbic acid is a plant-specific compound by itself, or if its presence is due to oxidation of ascorbic acid by processing [24].

High capsaicinoid content of certain peppers might delay fermentation (initiation), thus influencing vitamin C losses. It might seem as if low pungency varieties ferment slightly faster, leading to rapid pH drop, which could lead to the stabilization of some molecules, which under normal circumstances would be relatively unstable. This is evidenced in the case of moderately pungent pepper types, such as Cayenne and Bulgarian peppers, which present lower losses of dehydroascorbic acid 15.58% respectively 13.82% compared to highly pungent peppers (Habanero and Carolina Reaper) which present total losses of 28.91% and 29.31%. Pungent cultivars had a noticeable tendency to delay in fermentation and pH drop. This hypothesis however should be scientifically verified.

### 2.4. Phenolic Compounds

More than 10 phenolic compounds were identified from different subclasses (flavanols, hydroxybenzoic acid, and flavanones), that include catechin-derivatives, vanillic acid, ferulic acid-glucosides, naringenin-diglucoside, luteolin-apiosyl-glucoside, luteolin-(apiosyl-glucosyl-malonyl)-glucoside, luteolin-glucoside, myricetin, quercetin-(galloyl-caffeoyl-glucosyl)-rhamnoside, luteolin and naringin-malonate. Luteolin and quercetin derivatives are regarded as the main phenolic compounds, representing approximately 44% of phenolic compounds in the fresh samples. Values in fermented samples show similar values with some increase (46%). This is in accordance with articles that show values of approx. 41% [54]. The biodynamics of these compounds is also interesting, as results of the mentioned study underlines some interesting facts. For example, *C. chinense* peppers show values of 0.16% quercetin for fresh fruit (in immature fruits), levels that decrease as low as 0.01% (102.1 μg/g) in the mature fruits. Many studies present similar patterns, although the dynamics is not always as consistent as in the case of other compounds (carotenoids or capsaicinoids).

Interestingly, luteolin as described by Antonio (2008) [6], ranges between 0–151.1 μg/g in mature *C. chinense* fruit and 0–498.9 μg/g in mature *C. annuum* *fruits.* In our study, we could not detect luteolin in *C. chinense* fruits, but we recorded values between 7.92–42.23 μg/g for *C. annuum.* Thus, however we identified many derivatives (esters) of luteolin in all samples, with considerably higher values in case of slightly immature fruits (Bulgarian chilies). Data shows, that fermentation increases luteolin levels (probably due to hydrolysis of more complex derivatives). For *C. chinense* peppers, there is an increase from not detectible levels to a content of 5.08–42.79 μg/g. This tendency is also observed for the derivative luteolin-apiosyl-glucoside and luteolin glucoside, an increase through fermentation that seems to happen on the expanse of other complex glucosides, like luteolin-(apiosyl-glucosyl-malonyl)-glucoside.

Total phenolic content of the examined samples ranged between 36.89–195.43 mg/100 g for the fresh samples and 35.60–180.40 mg/100 g for the fermented samples. These values are very similar to those of Ana Carolina de Aguiar (2016), [49] who reported values ranging between 0.35–3.06 mg/g (gallic acid equivalent). Her slightly upper values can be explained again through sample preparation (peppers peeled, stems and seeds separated). She also reported maximum values in case of Naga Jolokia-type peppers. This, however, cannot explain a correlation between pungency and phenolic compounds. We couldn’t identify higher values for more pungent peppers, in fact, it is quite clear that total phenolic compounds are more predominant in immature fruits [6]. In our case, the mildest chilies (Bulgarian chili) showed values more than double for total phenolic compounds than the extremely pungent Carolina Reaper, although we need to specify that the first type was visually categorized as not fully matured.

Total average losses through fermentation are low and statistically insignificant (*p* > 0.05). Differences are evident amongst cultivars (Table 6), regarding different compounds, and they are statistically significant (*p* < 0.05). Also, a correlation is evident between total phenolic content and stage of fruit maturity. The Bulgarian cultivar contained the highest concentration of total phenols (195.43 ± 2.93 mg/100 g) with total losses of 7.69% through fermentation. Samples of these chilies were not properly colored (not fully matured). This tendency is highlighted by different articles, that suggest a decrease of total phenols at the late stage of full maturity to approx. 85% of the maximum content in earlier stages (green fruits), [6]. This tendency is also evidenced by the lower concentration of total phenolic compounds in chilies that were fully matured (Cayenne, Habanero, and Fatalii). These fruits were generally fully ripened and uniformly colored.

Average losses through fermentation are 11.63%, with minimum values in the case of Cherry peppers (0.19%) and maximum values for Fatalii (24.60%). Fatalii (yellow-type pepper) also showed (phenotypically) a more powerful discoloration of the paste, whereas red pastes were very stable regarding this aspect, showing no visible color loss during fermentation. No evident correlation can be seen between total phenolic content and pepper pungency (*p* > 0.05).

Amongst the different types of flavonoids, some tend to increase during fermentation, whereas others decrease in concentration. Catechin-derivatives present a general tendency towards increasing. The same tendency is available in the case of luteolin-apiosyl-glucoside and luteolin. Other compounds (vanillic acid, ferulic acid-glucosides, naringenin-diglucoside, luteolin-(apiosyl-glucosyl-malonyl)-glucoside present a lowering tendency.

Data analysis show statistically significant differences between pepper types in the same form, but they also indicate differences within same types in fermented and unfermented stages (*p* < 0.01). Through the 21-day fermentation period, catechin derivatives show an increasing trend to a maximum 481.70% in the case of the Cherry-type pepper. All types present similar tendencies, excepting Fatalii, which tends to reduce its content to approx. 92.82% of the initial concentration. Same general tendencies are to be observed in the case of luteolin-apiosyl-glucoside (*p* < 0.01). Values generally increase by 2–4 folds through fermentation excepting Carolina Reaper in the case of which a moderate decrease can be observed from 85.06 μg/g to 61.56 μg/g.

Other compound, like vanillic acid, naringenin-diglucoside and luteolin-(apiosyl-glucosyl-malonyl)-glucoside present an inverse trend. In all cases, differences are statistically significant (*p* < 0.05) both between different pepper types and between same pepper types in the different stages (unfermented/fermented). Vanillic acid is unevenly distributed amongst pepper samples, Carolina Reaper containing the highest amount 69.05 μg/g whereas Cayenne contains the least amount (9.27 μg/g). Fermentation strongly reduces vanillic acid content to 0% in most of the cases. Carolina Reaper and the Bulgarian types, however, show some remaining quantities (reduced by 2–3-fold compared to the initial values. All other compounds present variable trends. Some substances might increase in one pepper type during fermentation, whereas they might decrease in others. However, spontaneous fermentation of vegetables (in our case, chili peppers) represents a good method for bio-compound preservation and/or formation. Lacto-fermentation not only induces a series of biochemical changes in pepper paste that fortifies end products, but also assures a practical and low-cost way of preserving these valuable compounds by increasing the activity of H^+^ ions and lowering pH values [12].

Phenolic compounds tend to increase in dried or smoked pepper-fruits, and values may also increase if seeds are used in the extraction process, as some studies underline the presence of these compounds in the seeds [55].

### 2.5. Sugars and Acids

Sugars can be identified both in pericarp and placenta, higher amounts being evidenced in the pericarp (Table 7) [51]. Some studies suggest that glucose is the primary carbon source in peppers with percentage values ranging between 0.36–3.79%, followed by fructose (0.16–2.98%) [56]. Our results suggest that this might not always be the case, as we obtained higher values for fructose, in all samples. In fact our results are similar to those obtained by Jamiołkowska, 2016 [57] who presented fructose as the predominant sugar (2.74–2.98%), followed by glucose (2.42–2.60%) and sucrose (0.51–0.84%). However, it should be mentioned that in this study seeds were discarded.

Our study indicates that glucose content presents significant variation between different types of chilies (*p* < 0.05) within the same group. Results show that glucose content of our samples range between 1.29–1.94%, Cayenne showing maximum values (19.40 ± 0.70 mg/g). These values are in accordance with results of other studies.

As it is shown, *Capsicum annuum* species might contain slightly higher concentrations of glucose, as Carolina Reaper, for example, shows much lower values (12.86 ± 0.61 mg/g) compared to Cherry-type peppers (17.07 ± 0.15 mg/g). However, this cannot be taken as a general rule. For example a study conducted by Zamljen (2021) [51], indicates maximum values of total sugars for *C. chinense* and *C. frutescens* and much lower values for *C. annuum* species. In our experiment, although some cultivars (Fatalii and Carolina Reaper) showed lower values, Habanero peppers were amongst the leading samples regarding total sugar content (40.41 mg/100 g FW). Results can be debated, as different partitions (seeds, placenta, or pericarp) present great variability in each examined cultivar.

Fermenting bacteria utilise glucose as primary carbon source for metabolism. It is no surprise the fact that after the 21-day fermentation period, glucose values drop steeply in the interval of 0.04 ± 0.00 mg/g for Bulgarian and 2.58 ± 0.06 mg/g for Habanero respectively. Glucose drop is highly correlated with lactic acid and acetic acid formation. Lactic acid increases from average values of 0.02% to 1.13%. Similar tendencies are shown in the case of acetic acid, which increases from 0.00 mg/g in all samples to an overall average value of 4.05 mg/g (0.40%). Tendencies show a similar pattern in all cases regardless of pungency and capsaicin content. This might mean that regardless of capsaicin content, LAB are capable of fermenting glucose in all chili types, regardless of pungency (capsaicinoid content).

Fructose, however, presents quite a different tendency. Although there are statistical differences (*p* < 0.01) between different pepper types and same types in different stages, these differences are less significant than those in the case of glucose. This might be explained by the use of fructose as a secondary carbon source by fermenting bacteria, utilised only after total glucose metabolism. Fructose content ranges in the interval of 18.92 ± 0.60 mg/g for Fatalii and 23.72 ± 0.43 mg/g in the case of Cherry. After 21 days of fermentation values drop to 14.30 ± 0.05 mg/g (Fatalii) and 22.53 ± 0.18 mg/g (Cherry). It is interesting that in the case of Carolina Reaper, which presents lower glucose content in fresh fruits (12.86 ± 0.61 mg/g), fructose drop is more significant as it goes from 20.17 ± 0.29 mg/g to 10.72 ± 0.35 mg/g in 21 days, whereas such a significant change is not evident in other cases. This could be explained by the fact that low glucose content leads to rapid bacterial metabolism followed by a shift to secondary sugar sources.

Samples present significant differences (*p* < 0.01) in citric and malic acid content of the unfermented and fermented chilies. Differences, however, are not significant (*p* > 0.05) within the same category (fermented or unfermented version of the chili paste). However, in the case of unfermented chilies a pattern can be evidenced, that might indicate an inverse-linear decrees of malic acid compared with the increase of pungency. This pattern can be observed as levels of the compound drops from maximum values of 3.60 ± 0.13 mg/g (Cherry peppers) and 3.17 ± 0.07 mg/g (Bulgarian-type pepper) to 1.45 ± 0.02 mg/g (Fatalii) and 1.22 ± 0.12 mg/g (Carolina Reaper). Although these values might indicate a trend, this hypothesis might need further investigation.

Studies also indicate that, regardless of pepper cultivar, the predominant organic acid in fresh peppers is citric acid, followed by malic acid and succinic acid [58]. This was confirmed in our study. Total acid content of peppers in fresh peppers ranges between 135–708 mg/100 g [56].

Malic acid content of peppers ranges between 0.12–0.36% (FW). Fermentation during the three-week period leads to a significant drop to values between 0–0.09%. Losses are between 73.92–99.6% related to the initial quantities. This loss is generated by malolactic fermentation, and is usually caused by yeasts present in fermentable substrates [59]. *L. plantarum* species are also known to present similar activity, being capable of fermenting malic acid, with the formation of lactic acid and CO_2_ [60], as a secondary carbon source in the absence of glucose [61]. Both microorganisms are known to be found in fermenting vegetables, especially *L. plantarum*.

Citric acid presents a similar trend to that of malic acid. Maximum values of citric acid in the case of unfermented chili samples are attributed to the Bulgarian-type and Carolina Reaper with values of 8.18 ± 0.04 mg/g and 8.18 ± 0.15 mg/g. Minimum values are evident in the case of Fatalii and Habanero peppers with values of 4.73 ± 0.10 mg/g and 5.15 ± 0.15 mg/g. Average values of the examined samples are not significant (*p* > 0.05) between the examined pepper types. During fermentation however, losses are statistically significant (*p* < 0.05) and a drop in citric acid level is evident in all cases. Minimum and maximum values of citric acid levels in fermented chili paste reach 0.52 ± 0.02 mg/g and 1.95 ± 0.03 mg/g. Studies show that citrate is metabolized by LAB (*L. plantarum*; *L. brevis*; etc.) a process that leads to the formation of acetoin and diacetyl [62].

Succinic acid content of the examined material shows a slight increasing trend through fermentation. Average values increase from 0.02% to 0.11% by the 21st day. *C. chinense* species tend to show higher values of succinic acid in the fermented paste, although fresh peppers have quite similar values for the *C. annuum* and for *C. chinense* species. Succinic acid is a dicarboxylic acid generally regarded as the product of anaerobic microorganisms that perform mixed fermentations [63]. Although it presents a great interest for different industries, it is an undesirable compound in fermented foods, at it is associated with a bitter acidic taste.

Lactic acid and acetic acid represent the main acidic components in fermented chili, resulted by the metabolic activity of LAB and AAB (Acetic Acid Bacteria). Differences between fresh and fermented samples are statistically significant (*p* < 0.001). Lactic acid is predominant in fermented samples (10.32–13.06 mg/g), but traces can be identified in fresh samples also (0.08–0.32 mg/g). Acetic acid is absent in all fresh chili samples, but fermented samples present values between 3.22–4.85 mg/g, suggesting a strong oxidative (fermenting) activity of AAB only in fermented peppers. The ratio between the two compounds is between 2.3–3.2, considered an optimum for best flavour and aroma development [64].

### 2.6. Viability Assay

*Leuconostoc* species are hetero-fermentative bacteria that produce both acids (lactic and acetic) and ethanol during fermentation [65]. These are regarded as more active in the initial phase of fermentation, as their optimum growing pH is about 5.5. They adapt more rapidly to the substrate, acidifying the medium, thus creating optimum growing conditions for LAB growth. It is reported, that *lactobacilli* CFU values are usually better maintained in the last phase of fermentation, as they are more tolerant towards acidic stress [61].

In order to characterize microbial activity during fermentation, viability assay for LAB was conducted (MRS Agar) in parallel with isolation and quantification of *Leuconostoc* population on acetate agar (AA). One research article regarding microbial diversity for fermented jalapeño peppers indicate that *Lactobacillus* species were predominant, comprising for 56.65% of the total microbial population whereas *Leuconostoc citreum* and *Weisella cibaria* accounted for 25.75% and 17.60% in the study [66]. Many studies also indicate, that *Leuconostoc mesenteroides* plays an important role in the initiating phase of vegetable fermentation, being able to rapidly acidify the medium, thus having a rapid inhibitory effect over potential pathogens [64].

An open fermentation (recipients exposed to atmospheric oxygen) was first carried out on two types of pepper (Kapia and Cayenne) in order to pre-examine the relation between total LAB and *Leuconostoc* sp. in aerobic conditions. According to these results, spontaneous fermentation, involves an almost insignificant number of initial cells (1–2 × 10^2^ CFU/g), capable of rapid adaptation and proliferation in the substrate, reaching maximum population numbers in the first 24–48 h.

We presumed that capsaicin content of Cayenne-type pepper might partially inhibit the activity of LAB. However, plate-counts contradict our presumptions, in the sense that bacterial growth was intense on every pepper type. In fact, bacterial growth was more intense in the pungent mash (Cayenne). Bacterial cell viability showed a rapid drop after 24–48 h presenting a direct correlation with sugar metabolism. Both fructose and glucose were metabolised simultanously in a period of maximum 6–7 days. A correlation between *Leuconostoc* activity and total LAB was evident [67], indicating a symbiotic relation between different species of LAB. It is very probable that the more adapted, heterofermentative species (*Leuconostoc*) initiates the process, whereas other microorganisms such as *L. plantarum* may be involved in later stages, when microanaerobiosys is developed and pH is reduced. It is assumed that acetic acid and lactic acid, the predominant end products of spontaneous vegetable fermentation are mainly dependent on *Leuconostoc* and *Lactobacilli* activity [61].

**Figure 2:**
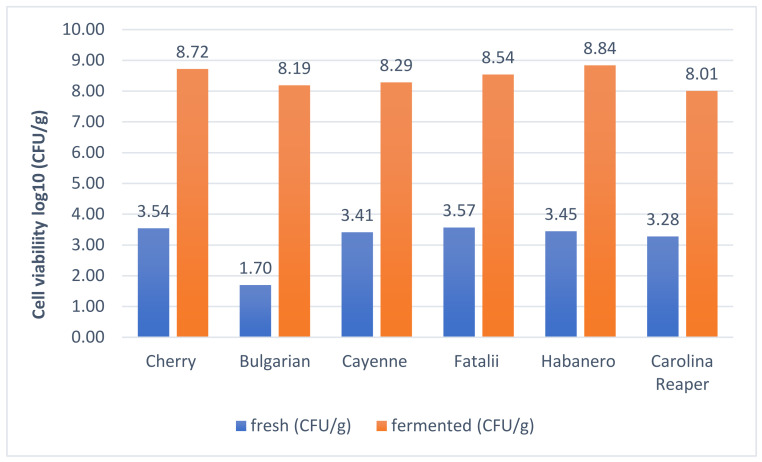
Viability assay of lactic acid bacteria in day 0 and day 21.

In anaerobic conditions (sealed recipients), fermentation presents a different pattern. As it is shown, in Figure 2, anaerobic spontaneous fermentation, tends to generate high amounts of CO_2_ in the first days (just like in the case of aerobic fermentation), but microbial activity is inhibited shortly after. Contrary to the first experiment, at the end of the fermentation period, significant amounts of fructose were still available. Glucose was almost totally metabolized (87–99%), but fructose was still present in between 53–95% of the initial values. LAB cell viability was high in this stage.

Fermented samples did not show significant differences in plate counts (*p* > 0.05). All samples fermented well and microbial colonies showed huge numbers. The total numbers of LAB were identified in between 8.01 log CFU/g (Carolina Reaper) and 8.84 log CFU/g (Habanero), whereas *Leuconostoc* cells were counted in between 6.72 log CFU/g (Carolina Reaper) and 7.72 log CFU/g (Cherry). Results are similar and differences are statistically insignificant (*p* > 0.05) between the evaluated samples, although they differ significantly in pungency (*p* < 0.001). Only Carolina Reaper showed significantly (*p* < 0.05) lower values (8.01 log CFU/g), in comparison with other types of pepper. This might indicate a low inhibition effect over LAB of peppers with exceptional pungency, although this hypothesis should be verified. Determinations of pH values also showed a delay in acidification of Carolina Reaper samples. However, after initiation of the process, pH drop continued according to similar patterns.

**Figure 3:**
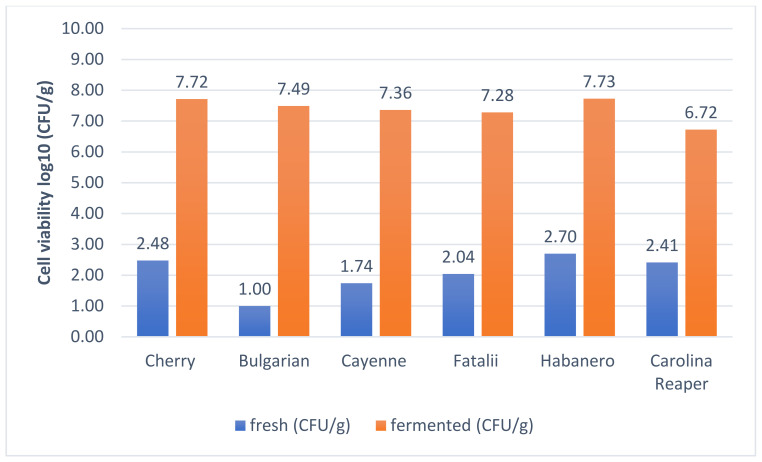
Viability assay of *Leuconostoc* bacteria in day 0 and day 21.

According to Figure 3, *Leuconostoc* strains were also present in the fermented mash, indicating a good survival kinetics, and good adaptation to the substrate. Cell counts indicated values between 6.72–7.73 log CFU/g, with maximum values reported in the case of Cherry- and Habanero-type peppers. Regardless of the fermentation method (aerobic/anaerobic), LAB and, especially, *Leuconostoc* cells proliferate and their variation presents a similar pattern, indicating that *Leuconostoc* might be the leading microorganism in this process, presenting a strong adaptation throughout the entire process.

In this study we tested different pepper samples in order to explore the possibilities of using fermented chili pastes as starter carriers for the production of certain fermented/cured products. LAB showed high plate counts in all fermented samples. However, further investigations should be considered in order to evaluate the survival kinetics of chili specific LAB on other substrates (vegetables or meat).

## 3. Materials and Methods

### 3.1. Sample Preparation

Six cultivars of chili peppers from two species were procured for analysis to be conducted: Bulgarian chili (*C. annuum*), Cayenne (*C. annuum*), Cherry (*C. annuum*), Habanero red (*C. chinense*), Fatalii (*C. chinense*), and Carolina Reaper (*C. chinense*). Selection of samples was based on color variety, pungency, and maturation. All samples were delivered from local growers and kept in similar conditions, avoiding contamination and degradation. The growing period of the chilies was characterized by low temperatures and humid weather, conditions that might have influenced some results.

**Figure 4:**
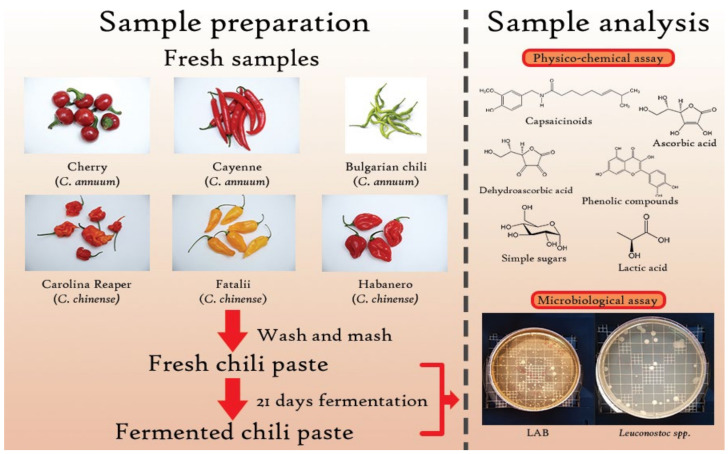
Experimental scheme—samples preparation and analysis.

Average amount of 1 kg of each pepper type was washed and mashed completely, including pericarp, placental tissue and seeds (only peduncle discarded), using a RobotCoop (3500 rpm) blender. Before fermentation, samples were taken for further analysis. Fermentation was carried out at 20 ± 1 °C without any seasoning or flavoring, and also no salt was added. After 21 days of fermentation, the same analyses were repeated and results were compared, as described in Figure 4.

L-ascorbic acid, capsaicin and dehydro-capsaicin, gallic acid and rutin standards were procured from Sigma-Aldrich (St. Louis, MO, USA); methanol, metaphosphoric acid, and acetonitrile were obtained from Merck (Darmstadt, Germany). All reagents were of analytical grade.

### 3.2. Sample Analysis

#### 3.2.1. Ascorbic Acid and Dehydroascorbic Acid Quantification (HPLC-DAD-ESI^+^)

0.5 g of the examined samples were weighed and 5 mL aqueous solution of metaphosphoric acid (3%) + acetic acid (8%) was added to each sample. Each test tube was vortexed for 1 min (Heidolph-Reax, Heidolph-Instruments, Schwabach, Germany), sonicated for 30 min (Elmasonic E 15 H, Singen, Germany), and centrifuged (8000 rpm/10 min, at 4 °C) using a Eppendorf AG 5804 centrifuge (Hamburg, Germany). Supernatant of each extract was filtered (Chromafil Xtra nylon 0.45 µm) and 20 µL were injected in the HPLC system. Identification of the compounds was achieved according to retention time, UV-Vis and mass spectra. Retention time was 3.15 min for ascorbic acid and 4.08 min for dehydroascorbic acid.

For quantification of the examined compounds, a calibration curve was made by injecting five different concentrations of standard solutions (ascorbic acid), procured from Sigma-Aldrich.

HPLC analysis was conducted by using the HPLC Agilent 1200 system equipped with a quaternary pump, autosampler, UV-Vis coupled with photodiode (DAD) and mass detector (MS) Agilent 6110 (Agilent Technologies, Santa Clara, CA, USA). For the separation of the compounds, an Eclipse XDB C18 column was used (size: 4.6 × 150 mm). Mobile phase conceived of water/acetonitrile solution 95/5 (*v*/*v*) and 1% formic acid. Time flow rate was 0.5 mL/min, 10 min, 25 °C. Spectral values were registered at wavelength ranges between 200–400 nm. Chromatograms were identified at λ = 240 nm. Electrospray ionization (ESI) technique was used with the following parameters: capillary voltage (3000 V), temperature (300 °C), nitrogen flow rate (7 L/min), *m*/*z* (100–600, full-scan). Interpretation of the results was done by using the Agilent ChemStation software (Rev B. 04.02 SP1, Palo Alto, CA, USA).

#### 3.2.2. Phenolic Compounds and Capsaicinoids Extraction

Extraction of the compounds was made by adding 5 mL methanol + 1% HCl acid to 1 g of each sample. Samples were vortexed for one minute (Heidolph Reax), sonicated for 30 min (Elmasonic E 15 H), macerated for 24 h at 4 °C and centrifuged at 8000 rpm/10 min at room temperature (Eppendorf AG 5804, Hamburg, Germany). The supernatant was filtered (Chromafil Xtra nylon; 0.45 μm). 20 μL of each extract were injected in the HPLC system. Chromatograms were registered at λ = 280 nm for capsaicinoids and λ = 340 nm for phenolic compounds. Identification of capsaicinoids was made according to retention time, UV-Vis spectra and mass. Five concentrations of capsaicin standards (98.5% purity) were injected for capsaicinoid quantification.

For quantification of phenolic compounds (hydroxybenzoic acids, flavanols, and flavanones) calibration was made by using five different concentrations of gallic acid, as for flavones and flavonols, calibration was made with rutin. All standards were acquired from Sigma-Aldrich.

For HPLC analysis Agilent 1200 system equipped with a quaternary pump, autosampler, UV-Vis coupled with diode-array detection (DAD) and mass detector (MS) Agilent 6110 (Agilent Technologies, Santa Clara, CA, USA) were used. The same column was used as in the case of ascorbic acid, mobile phases consisting of A (H_2_O + 0.1% acetic acid) and B (acetonitrile + 0.1% acetic acid). Gradients (% B) were as presented: 0 min, 5% B; 0–2 min, 5% B; 2–18 min, 5–40% B; 18–20 min, 40–90% B; 20–24 min, 90% B; 24–25 min, 90–5% B; and 25–30 min, 5% B.

Electrospray ionization (ESI) technique was used with the following parameters: capillary voltage (3000 V), temperature (350 °C), nitrogen flow rate (7 L/min), *m*/*z* (120–1200, full-scan). Interpretation of the results was done by using the Agilent ChemStation software.

#### 3.2.3. Microbiological Analysis

Bacterial counts from fresh and fermented pepper sauces were performed at day 0 and day 21 of fermentation. Aseptically collected samples were suspended in physiological serum (0.9% *w*/*v* of NaCl), afterwards being homogenised for 30 s. Serial dilutions were carried out, in sterile physiological serum, using 1:9 ratio and plated according to the surface plate technique on different media for viable counts. LAB were counted on MRS agar after 48 h incubation at 37 °C, whereas *Leuconostoc* was counted on acetate agar after 72 h incubation at 25 °C.

#### 3.2.4. Statistical Interpretation

Statistical interpretation of the results was conducted using the Microsoft Exel 2010 program. Recorded values were compared using the ANOVA test (two factors with replication), Microsoft Excel 2016, as two variants were involved: types of pepper (six types from two species) and way of usage (unfermented and fermented samples). The results are expressed as means values ± standard deviations of three independent (*n* = 3) assays.

## 4. Conclusions

Spontaneous fermentation of chili peppers represents a technologically easy and low-cost method for preservation and bio-fortification. Fermentation increases capsaicinoid content, probably due to better extraction of the macerated samples. Newly formed conditions in the mash (lack of oxygen, pH < 4) prevent losses of ascorbic acid through stabilization of the molecules. Some phenolic compounds seem to be metabolized, whereas others are synthesized. LAB are the predominant microorganisms responsible for sugar metabolism and acid formation. Viability assay shows that LAB survive in fermented pepper paste and are present in large numbers, even after 21 days.

Rethinking production towards more natural and traditional ways is required as a response to the failure of modern society to deal with overprocessed foods and unhealthy nutritional habits. Fermented pepper paste might show a good potential in the production of cured meat or vegetable products, as fermentation is considered to be one of the most primitive thus safest ways of preserving foods without added chemical elements. However, adaptation of spontaneous microflora to other substrates (e.g., meat) should be further investigated.

## Figures and Tables

**Table 1:** Partition of different components of *Capsicum* fruits (%).

Sample	Pericarp (%)	Placenta (%)	Seeds (%)
Cherry	75.42 ± 1.47	11.91 ± 0.25	12.67 ± 1.28
Bulgarian	90.68 ± 3.70	6.49 ± 3.12	2.82 ± 1.04
Cayenne	88.35 ± 1.06	8.90 ± 0.53	2.74 ± 1.37
Fatalii	80.61 ± 2.14	8.63 ± 1.50	10.75 ± 1.22
Habanero	86.54 ± 2.85	8.99 ± 1.61	4.47 ± 1.25
Carolina Reaper	86.27 ± 5.89	8.08 ± 2.36	5.65 ± 4.26

Values (%) represent means ± standard deviation of mean for 3 replicates.

**Table 2:** Average humidity of fresh and fermented pepper samples (%).

Sample	Fresh	Fermented
Cherry	85.48 ± 2.06	86.96 ± 0.59
Bulgarian	86.87 ± 1.75	87.98 ± 0.86
Cayenne	88.14 ± 1.02	90.34 ± 1.25
Fatalii	84.64 ± 0.40	88.30 ± 1.09
Habanero	88.58 ± 0.60	89.34 ± 0.39
Carolina Reaper	86.87 ± 0.02	89.06 ± 0.62

Values (%) represent means ± standard deviation of mean for 3 replicates.

**Table 3:** Distribution of different capsaicinoids in tested samples (μg/g).

	Sample	n-DHC (μg/g)	Caps. (μg/g)	DHC (μg/g)	h-DHC (μg/g)	Total Capsaicinoids (μg/g)
Fresh	Cherry	9.62 ± 0.37	248.25 ± 2.01	198.60 ± 7.96	22.43 ± 1.72	478.90 ± 9.06
	Bulgarian	5.46 ± 0.07	88.41 ± 2.96	58.31 ± 2.88	8.98 ± 0.12	161.15 ± 5.96
	Cayenne	5.14 ± 0.03	99.63 ± 5.18	79.13 ± 1.31	25.96 ± 0.90	209.84 ± 5.49
	Fatalii	117.56 ± 7.40	2365.16 ± 148.67	1317.76 ± 12.00	286.69 ± 6.11	4087.16 ± 154.61
	Habanero	21.47 ± 1.41	501.93 ± 3.60	277.40 ± 1.22	39.41 ± 0.50	840.21 ± 2.31
	Carolina Reaper	163.69 ± 9.87	9574.64 ± 269.15	6627.81 ± 177.99	191.87 ± 5.88	16,558.01 ± 313.89
Fermented	Cherry	26.28 ± 0.92	262.02 ± 22.14	259.46 ± 3.07	85.53 ± 2.22	633.29 ± 24.30
	Bulgarian	23.07 ± 1.01	104.11 ± 2.72	93.54 ± 1.00	52.86 ± 2.13	273.58 ± 4.83
	Cayenne	19.87 ± 1.02	152.16 ± 2.55	120.77 ± 4.87	35.88 ± 1.85	328.67 ± 6.62
	Fatalii	109.56 ± 3.75	2660.16 ± 30.01	1495.21 ± 40.88	343.06 ± 8.55	4607.98 ± 82.21
	Habanero	29.48 ± 2.41	547.74 ± 15.53	403.60 ± 3.16	77.52 ± 1.66	1058.33 ± 21.99
	Carolina Reaper	212.05 ± 15.21	9798.22 ± 159.50	6769.07 ± 60.84	1222.62 ± 22.61	18,001.96 ± 83.94

Values (μg/g) represent means ± standard deviation of mean for 3 replicates.

**Table 4:** Pungency of the studied samples in their fresh and fermented forms (SHU).

Sample	Fresh	Fermented
Cherry	51,156 ± 1224	71,463 ± 3304
Bulgarian	18,844 ± 723	31,835 ± 724
Cayenne	26,391 ± 946	50,279 ± 1489
Fatalii	406,524 ± 17,511	601,810 ± 10,611
Habanero	113,871 ± 825	151,563 ± 3146
Carolina Reaper	1,999,058 ± 55,578	2,536,507 ± 35,263

Values (SHU) represent means ± standard deviation of mean for 3 replicates.

**Table 5:** Average values for ascorbic acid content in different pepper samples (μg/g).

	Pepper Types	Ascorbic Acid	Dehydroascorbic Acid	Total Ascorbic Acid
Fresh	Cherry	159.41 ± 1.52	504.09 ± 1.99	663.50 ± 0.69
	Bulgarian	224.80 ± 4.44	420.77 ± 17.14	645.58 ± 21.47
	Cayenne	954.41 ± 3.49	391.43 ± 10.25	1345.84 ± 9.26
	Fatalii	337.56 ± 16.55	416.69 ± 15.28	754.25 ± 28.97
	Habanero	360.93 ± 10.77	423.71 ± 21.34	784.64 ± 28.37
	Carolina Reaper	148.93 ± 1.73	417.63 ± 12.16	566.56 ± 13.15
Fermented	Cherry	141.70 ± 1.52	384.30 ± 13.36	526.00 ± 13.98
	Bulgarian	202.69 ± 2.56	362.61 ± 16.08	565.30 ± 18.49
	Cayenne	747.75 ± 20.62	330.44 ± 10.41	1078.18 ± 25.79
	Fatalii	297.32 ± 11.53	305.08 ± 13.24	602.40 ± 20.58
	Habanero	349.83 ± 11.07	301.20 ± 11.44	651.02 ± 17.12
	Carolina Reaper	136.35 ± 6.33	295.23 ± 19.94	431.58 ± 24.97

Values (μg/g) represent means ± standard deviation of mean for 3 replicates.

**Table 6:** Content of phenolic compounds before and after fermentation (µg/g).

	Sample	Catechin- Derivatives	Vanillic Acid	Ferulic Acid Glucoside	Naringenin-Diglucoside	Luteolin- Apiosyl- Glucoside	Quercetin- Glucoside	Luteolin-(Apiosyl- Glucosyl-Malonyl)- Glucoside	Luteolin-Glucoside	Myricetin	Quercetin-(Galloyl- Caffeoyl-Glucosyl)- Rhamnoside	Luteolin	Naringin- Malonate	TPC
Fresh	Cherry	44.22 ± 1.35	33.51 ± 3.26	114.82 ± 4.22	106.82 ± 2.84	50.37 ± 2.18	32.37 ± 3.65	208.83 ± 7.31	224.94 ± 5.04	12.85 ± 1.02	67.62 ± 1.51	15.31 ± 1.53	123.92 ± 2.96	1035.57 ± 20.16
	Bulgarian	116.93 ± 3.54	55.66 ± 5.27	121.64 ± 6.03	81.39 ± 1.81	68.19 ± 2.54	137.56 ± 4.94	254.51 ± 5.00	568.76 ± 7.22	112.73 ± 6.18	86.39 ± 3.56	42.23 ± 2.55	308.31 ± 6.37	1954.30 ± 29.31
	Cayenne	74.10 ± 2.95	9.27 ± 0.27	53.41 ± 2.46	15.96 ± 1.70	19.67 ± 1.00	31.23 ± 3.84	58.15 ± 2.21	54.73 ± 0.64	28.77 ± 3.19	21.19 ± 1.08	7.92 ± 0.80	109.79 ± 5.83	484.18 ± 12.65
	Fatalii	169.87 ± 4.21	23.25 ± 1.84	24.79 ± 1.86	92.39 ± 1.53	6.02 ± 0.90	6.59 ± 0.40	33.51 ± 0.87	39.76 ± 2.35	12.47 ± 1.38	13.04 ± 0.97	0.00 ± 0.00	88.38 ± 2.74	510.06 ± 12.78
	Habanero	112.17 ± 2.00	17.89 ± 0.29	14.55 ± 2.00	23.54 ± 2.30	16.26 ± 2.01	19.86 ± 2.45	50.37 ± 2.02	50.94 ± 2.77	13.61 ± 1.95	14.36 ± 1.33	0.00 ± 0.00	35.29 ± 0.63	368.85 ± 10.36
	Carolina Reaper	154.40 ± 5.14	69.05 ± 1.77	62.51 ± 3.01	98.04 ± 4.27	85.06 ± 1.96	82.22 ± 4.04	110.08 ± 5.46	111.97 ± 6.05	58.15 ± 2.54	29.15 ± 1.01	0.00 ± 0.00	62.06 ± 1.92	922.68 ± 11.52
Fermented	Cherry	212.99 ± 2.64	0.00 ± 0.00	21.00 ± 2.97	55.96 ± 1.02	208.45 ± 11.09	60.61 ± 2.96	38.24 ± 2.09	218.50 ± 7.49	6.97 ± 0.88	17.40 ± 1.28	41.66 ± 0.63	150.87 ± 5.22	1032.65 ± 15.58
	Bulgarian	294.93 ± 3.45	15.81 ± 1.20	50.57 ± 5.07	62.65 ± 4.11	229.30 ± 7.40	96.81 ± 4.05	113.49 ± 6.50	591.13 ± 7.44	33.89 ± 2.93	64.59 ± 2.22	46.21 ± 2.01	204.66 ± 5.15	1804.03 ± 15.54
	Cayenne	115.29 ± 5.01	0.00 ± 0.00	6.97 ± 0.20	82.13 ± 2.55	42.79 ± 3.83	18.15 ± 0.33	14.74 ± 1.71	61.18 ± 1.20	5.26 ± 0.25	11.90 ± 2.02	12.85 ± 1.56	44.21 ± 2.63	415.49 ± 17.60
	Fatalii	157.67 ± 2.25	0.00 ± 0.00	7.16 ± 0.18	4.36 ± 1.04	11.90 ± 0.54	20.62 ± 0.28	15.31 ± 2.01	45.07 ± 2.65	17.96 ± 1.11	29.53 ± 0.52	5.08 ± 0.19	69.94 ± 5.42	384.59 ± 10.09
	Habanero	145.93 ± 4.06	0.84 ± 0.08	12.09 ± 1.40	16.55 ± 2.26	30.28 ± 0.35	17.21 ± 1.26	18.91 ± 2.06	42.98 ± 3.51	15.50 ± 1.00	16.45 ± 1.27	7.16 ± 0.14	33.80 ± 3.40	357.71 ± 8.40
	Carolina Reaper	185.63 ± 5.50	35.59 ± 2.47	32.75 ± 2.89	4.56 ± 0.21	61.56 ± 3.33	46.20 ± 3.29	31.61 ± 2.57	112.78 ± 2.49	28.77 ± 2.25	30.47 ± 0.71	42.79 ± 2.98	127.78 ± 10.28	740.50 ± 14.10

**Table 7:** Evaluation of sugar and acid content of different pepper samples (mg/g).

	Sample	Glucose	Fructose	Lactic Acid	Acetic Acid	Malic Acid	Citric	Succinic
Fresh	Cherry	17.07 ± 0.15	23.72 ± 0.43	0.28 ± 0.01	0.00 ± 0.00	3.60 ± 0.13	7.22 ± 0.03	0.21 ± 0.00
	Bulgarian	16.47 ± 0.46	23.11 ± 0.34	0.32 ± 0.01	0.00 ± 0.00	3.17 ± 0.07	8.18 ± 0.04	0.22 ± 0.01
	Cayenne	19.40 ± 0.70	23.25 ± 0.07	0.17 ± 0.01	0.00 ± 0.00	2.92 ± 0.08	6.11 ± 0.20	0.18 ± 0.01
	Fatalii	13.55 ± 0.53	18.92 ± 0.60	0.08 ± 0.01	0.00 ± 0.00	1.45 ± 0.02	4.73 ± 0.10	0.16 ± 0.01
	Habanero	17.79 ± 0.37	22.62 ± 0.18	0.10 ± 0.01	0.00 ± 0.00	1.53 ± 0.08	5.15 ± 0.15	0.20 ± 0.01
	Carolina Reaper	12.86 ± 0.61	20.17 ± 0.29	0.08 ± 0.00	0.00 ± 0.00	1.22 ± 0.12	8.18 ± 0.15	0.20 ± 0.01
Fermented	Cherry	0.34 ± 0.10	22.53 ± 0.18	11.33 ± 0.18	4.37 ± 0.02	0.94 ± 0.02	1.95 ± 0.03	0.66 ± 0.02
	Bulgarian	0.04 ± 0.00	19.45 ± 0.10	13.06 ± 0.13	4.85 ± 0.05	0.02 ± 0.00	1.48 ± 0.11	0.91 ± 0.01
	Cayenne	0.86 ± 0.06	19.10 ± 0.12	12.15 ± 0.07	4.08 ± 0.04	0.34 ± 0.01	1.12 ± 0.12	0.56 ± 0.01
	Fatalii	2.34 ± 0.32	14.30 ± 0.05	10.34 ± 0.01	3.22 ± 0.01	0.18 ± 0.01	0.64 ± 0.02	1.35 ± 0.03
	Habanero	2.58 ± 0.06	17.25 ± 0.58	10.70 ± 0.05	3.29 ± 0.10	0.25 ± 0.01	0.52 ± 0.02	1.41 ± 0.01
	Carolina Reaper	0.32 ± 0.01	10.72 ± 0.35	10.32 ± 0.28	4.47 ± 0.12	0.01 ± 0.00	0.98 ± 0.06	1.92 ± 0.09

Values (mg/g) represent means ± standard deviation of mean for 3 replicates.

## Abbreviations

LAB	Lactic Acid Bacteria
UCP	Unfermented Chili Pastes
FCP	Fermented Chili Pastes
FW	Fresh Weight
DHC	Dihydrocapsaicin
n-DHC	Nordihydrocapsaicin
h-DHC	Homodihydrocapsaicin
DW	Dry Weight
SHU	Scoville Heat Units
ppm	parts per million
AAB	Acetic Acid Bacteria
